# Nursing Affairs in Ireland

**Published:** 1917-12-15

**Authors:** 


### NURSING AFFAIRS IN IRELAND

### Ideals.

What do Irish Nurses hope to achieve? This is a question which every trained member of the profession ought to put to herself with all seriousness. At the moment, owing to the multitude of advisers, the average work-a-day nurse is in a condition of bewilderment. Possibly the same set of circumstances may apply to nurses in Great Britain. In Ireland there is no room for doubt that the nurse is being treated as a pawn in a game played for leadership, to which her interests are entirely subordinate. It is a case of join this Board or that League, pay your guinea, and leave the rest to us, whereas what is really wanted is a clear statement of attainable ideals, the elimination of impossible achievements, and then complete and whole-hearted co-operation in order to ensure success. Before the College of Nursing pitched its tent in Ireland the nursing profession lived from day to day in peaceful chaos. A little coterie of matrons held their conversaziones, discussed the weather and the prospects of a Bill which they had nursed with care for a quarter of a century, gathered in such guineas and half-crowns as they could realise in order to conduct their academic business—and nothing more. With the advent of the College they awoke, rubbed their eyes, and, fearing obliteration, sought about for popular war cries and vote-securing shibboleths. "Lay control," "English" aggression, "a State-protected profession," "the pauperisation of nurses"—all these utterly meaningless war cries are being exploited with a nod and a wink about this person and that, and then follows the inevitable request for a guinea. Through all the din the nurse sits cold, unmoved, and embarrassed. The rank and file are experiencing for the first time in their lives the sensation of being appealed to, and, of course, in a country like Ireland, torn with political dissension, Patriotism is a call that the crafty leaders do not fail to exploit.

There are two outstanding facts which must be borne home to the plain woman who is earning her living by nursing and who has not the distinction of being a leader. The first is that there are certain possibilities which may be realised, and that these may be clearly defined. The second is that without the complete and concerted action of the entire profession failure must ensue. Faction and chaos are twin brethren, and although Donnybrook Fair is no more than a tradition, the soul of Donnybrook is still marching along. I have no hesitation in affirming that the rank and file are not yet infected by the sects. As a body they stand aloof, keep their guineas in their purses, and watch the battle from afar. They are right. I hold the view that no nurse ought to pay a guinea to any institution or enrol as a member thereof until she clearly realises the issues. In order to arrive at this stage the nurse must begin to think for herself, and she must realise that she may have to do so in certain instances entirely independently of what her matron may wish. If, for example, that great lady comes to her and says, "Of course you will join the So-and-so Association," she must be sufficiently frank and diplomatic to stand aloof unless she is herself convinced that she is not thereby supporting the cause of faction and chaos. I am aware, I regret to observe, that in Dublin the omnipotent matron sometimes uses her influence to gather the nurses of her hospital into her fold, as a shepherd gathers his sheep, without argument or reason, and, if they assent, the subscription fees are deducted from their salaries when pay-day arrives. It is a very simple recruiting artifice, and almost invariably successful. But from the nurse's point of view it is tragic, and, although this is a strong word, it is used without exaggeration. The plight of the Irish nurse is much worse than even she herself realises. As a probationer she pays a large sum to be a hewer of wood and a drawer of water. She works afterwards at a salary that a well-qualified domestic servant would scorn. She has no holiday home, no pension scheme, no representative council to protect her in professional difficulty—not even a club where she can foregather with the others of her sisterhood. She is, in a word, an isolated, ill-paid, and hard-worked woman—a voice crying in the wilderness.

Is it not possible, I ask, in these circumstances, to separate the true from the false, and to segregate faction and faction-mongers from that which makes for progress and material betterment? I believe this to be possible, always provided that the individual nurse is willing to think and to act for herself, and to refuse to be influenced or coerced by the press-gang matron. Hospital Boards must see that nurses' salaries are paid to them without deduction, and public meetings for the discussion of nursing affairs must be held until the air is cleared of doubt and mystery. The trained nurse should bear in mind that her hour of achievement may be at hand. The camp of faction must be avoided and a complete union of British nurses sought after whose guiding star will be the immediate and ultimate benefit of the profession.

IERNE.

